# Quantification of pulsed electric field for the rupture of giant vesicles with various surface charges, cholesterols and osmotic pressures

**DOI:** 10.1371/journal.pone.0262555

**Published:** 2022-01-13

**Authors:** Md. Kabir Ahamed, Marzuk Ahmed, Mohammad Abu Sayem Karal

**Affiliations:** Department of Physics, Bangladesh University of Engineering and Technology, Dhaka, Bangladesh; East China Normal University School of Life Sciences, CHINA

## Abstract

Electropermeabilization is a promising phenomenon that occurs when pulsed electric field with high frequency is applied to cells/vesicles. We quantify the required values of pulsed electric fields for the rupture of cell-sized giant unilamellar vesicles (GUVs) which are prepared under various surface charges, cholesterol contents and osmotic pressures. The probability of rupture and the average time of rupture are evaluated under these conditions. The electric field changes from 500 to 410 Vcm^-1^ by varying the anionic lipid mole fraction from 0 to 0.60 for getting the maximum probability of rupture (i.e., 1.0). In contrast, the same probability of rupture is obtained for changing the electric field from 410 to 630 Vcm^-1^ by varying the cholesterol mole fraction in the membranes from 0 to 0.40. These results suggest that the required electric field for the rupture decreases with the increase of surface charge density but increases with the increase of cholesterol. We also quantify the electric field for the rupture of GUVs containing anionic mole fraction of 0.40 under various osmotic pressures. In the absence of osmotic pressure, the electric field for the rupture is obtained 430 Vcm^-1^, whereas the field is 300 Vcm^-1^ in the presence of 17 mOsmL^-1^, indicating the instability of GUVs at higher osmotic pressures. These investigations open an avenue of possibilities for finding the electric field dependent rupture of cell-like vesicles along with the insight of biophysical and biochemical processes.

## 1 Introduction

Biomembranes are selectively permeable barrier which separates the interior and exterior of an organism [1,2]. Various types of equivalent circuit of biomembranes are considered by different groups [3–5]. Large electric potential is rare in living organisms, except in the region of cell membrane which is about—70 mV [6]. This potential is crucial for the transport of ions either into the lumen or out from the cells [7,8]. Because of least invasive nature and flexibility to adjust the strength, the external electric field has long been recognized as an excellent external agent for disrupting the cells [9–11]. Synthetic lipid vesicles such as giant unilamellar vesicles (GUVs) are being used as an attractive tool in soft matter research for the mimicking of cells since a few decades [12,13]. Lipid membranes of GUVs are significantly affected by the external electric field due to the charges on their polar head groups and the limited permeability of the hydrophobic tail to the solvent. Considering the electrical environment of cells or vesicles, external electric field can interact with the lipid membranes in various ways by influencing electrical properties such as electrical conductivity [14,15], membrane capacitance [16,17], transmembrane voltage [18,19] etc. Several types of phenomena can occur depending on the nature of electric field, for example, permeabilization [20–22], fusion [23,24], electrophoresis [25] and deformation [26,27]. Among them, electroporation is a vastly accepted technique, responsible for the generation of pores in vesicles when they are subjected to large electric field that can promptly increase the permeability of membranes. Sufficiently high electric potential applied to the membrane promotes stretching instability, which leads to the pore formation since the applied electric field increases membrane bending stiffness and lowers membrane tension [28]. The process of electroporation is actually twofold [29]. Firstly, a molecular rearrangement of the lipid occurs within the membrane bilayer induced by electrostatic forces and secondly, the Maxwell stress tensor starts to rise from the applied electric field. There is an increase in membrane current and membrane dynamic conductance with the ascending transmembrane voltage [30]. Now-a-days, electroporation is commonly used in many areas of biomedical, biotechnology, bioengineering, and medicine, for applications such as cell fusion [31,32], electrochemotherapy [11,33,34], gene transfer [35,36], cancer treatment along with localized tumor ablation [34,37,38], food processing [39,40] and so on. Based on the electric field parameters, this permeabilization is either reversible or irreversible [41–43]. A limited number of studies on membrane permeabilization due to electric field have already been performed as an attempt to improve and model the effects of electric field parameters, such as intensity and duration [44–46]. In the molecular dynamic (MD) simulations, it is evident that the transmembrane voltage is generated either by a direct electric field or by charging of membranes [47,48]. Continuum-level description of membrane electroporation from MD simulations has been compared to the experimental measurements on model lipid systems [49,50]. There is a general consideration that transmembrane voltage, induced by electric field, promotes rupture of GUVs. So far, there is no cognizant report on the quantification of electric field for vesicle rupture under various conditions such as surface charges and cholesterol contents of the membranes, and osmotic pressures in the vesicles. Hence, the experimental measurements of the values of external electric fields and the average times for the rupture of GUVs under those conditions are indispensable.

## 2 Materials and methods

### 2.1 Chemicals and reagents

1,2-dioleoyl-*sn*-glycero-3-phospho-(1′-*rac*-glycerol) (sodium salt) (DOPG) and 1, 2-dioleoyl-*sn*-glycero-3-phosphocholine (DOPC) were purchased from Avanti Polar Lipids Inc. (Alabaster, AL). Bovine serum albumin (BSA), 1,4-Piperazinediethanesulfonic acid (PIPES), Ethylene glycol-bis(2-aminoethylether)-*N*,*N*,*N*′,*N*′-tetraacetic acid (EGTA) and calcein were purchased from Sigma-Aldrich (Germany). Cholesterol (i.e., Chol) was purchased from WAKO pharmaceuticals (Japan).

### 2.2 Preparation of GUVs at various conditions

The anionic charged GUVs were prepared in a physiological buffer (10 mM PIPES, 150 mM NaCl, pH 7.0, 1mM EGTA) and the neutral GUVs were prepared in MilliQ water using the natural swelling method [51]. Here, the method is described briefly. A mixture of 1 mM DOPG and DOPC (about 200 μL) or DOPG, DOPC and Chol were taken into a glass vial and dried with a gentle flow of N_2_ gas for producing thin and homogeneous lipid films. By keeping the vial in a vacuum desiccator for 12 hours, the residual chloroform in the film was removed. Then, 20 μL MilliQ water was added into the vial and pre-hydrated at 45–47°C for 8 minutes. After pre-hydration, the sample was incubated with 1 mL of buffer containing 0.10 M sucrose for about 3 hours at 37°C. To prepare water-soluble fluorescent probe (calcein) containing GUVs, vesicles were incubated in the buffer with 0.10 M sucrose containing 1 mM calcein. The incubated GUV suspension (unpurified) was centrifuged at ~ 13,000×g (here g is the acceleration due to gravity) for ~ 20 minutes at ~ 20°C for removing the multilamellar vesicles and lipid aggregates as these elements were sedimented at the bottom of eppendorf tubes. After centrifugation, supernatant was collected and purified by the membrane filtering method [52,53].

To prepare the GUVs with different surface charges, the DOPG mole fractions (*X*_DOPG_) were considered 0.60, 0.40, 0.20, 0.10, 0. Hence, the samples were DOPG/DOPC (60/40, here 60/40 indicates molar ratio), DOPG/DOPC (40/60), DOPG/DOPC (20/80), DOPG/DOPC (10/90) and DOPG/DOPC (0/100)-GUVs. To prepare cholesterol (Chol) containing membranes, the samples DOPG/DOPC/Chol (60/40/0), DOPG/DOPC/Chol (46/39/15), DOPG/DOPC/Chol (43/28/29) and DOPG/DOPC/Chol (40/20/40)-GUVs were prepared in the same buffer. The corresponding cholesterol mole fraction in these samples were 0, 0.15, 0.29 and 0.40. By considering the surface areas of DOPG and cholesterol [54–59], the surface charge density of these cholesterol containing membranes were obtained almost similar (~ - 0.15 Cm^-2^). For performing the osmotic pressure experiments, DOPG/DOPC (40/60)-GUVs were prepared in the buffer under various osmotic pressures (Π). The osmolarity of the sucrose solution inside the GUVs was $C_{\mathrm{in}}^{0}$ = 388 mOsmL^-1^, whereas the osmolarities of the glucose solution outside the GUVs were *C*_out_ = 388, 375 and 371 mOsmL^-1^. Hence, the corresponding osmolarity difference between the inside and outside of GUVs were $\Delta C^{0}=C_{\mathrm{in}}^{0}-C_{\mathrm{out}}$ = 0, 13 and 17 mOsmL^-1^. Due to the osmolarity difference between sucrose and glucose solutions, vesicles became swell as water molecules of glucose solution passed into the inside of GUVs through membranes. The osmotic gradient creates lateral tension in the membranes of GUVs. The detail procedure to apply the Π in GUVs has been described in our recent paper [60]. To avoid the strong adhesion between the GUVs and the surface of glass slide, the chamber was coated with 0.10% (w/v) BSA dissolved in the same buffer.

### 2.3 Model to apply the electric field on vesicle using COMSOL simulation

To estimate the electric potential gradient and the distribution of current density on a vesicle, the finite element method-based software COMSOL Multiphysics was used. The simulation was used to investigate transport processes in various model systems [61]. The parameters of the model are summarized in Table 1. The electrodes were modeled as two rectangular plates placed at the left and right of the center of geometry. The mesh size was refined until there was less than 5% difference in electric field, resulting an extremely fine mesh setting. The total number of degrees of freedom solved in the model was 153669. The model describes electric field strength and current density in a spherical shaped vesicle. The dynamic finite-element model for efficient modelling of electric currents in electroporated tissue has been described elsewhere [62]. The time dependent electric current density equation is as follows:

$$J=(\lambda +\varepsilon_{0}\varepsilon_{r}\frac{\partial }{\partial t})E+J_{e}$$ (1)

where, *λ* is an electrical conductivity of the system and *J*_e_ is the charge density, *ε*_0_ is the permittivity of free space and *ε*_r_ is the relative permittivity. The electric field (*E*) is the gradient of electric potential (*V*), i.e., *E* = -∇*V*.

**Table 1:** The parameters, materials and values for COMSOL simulation.

Parameters	Materials	Symbol	Values
Vesicle	Radius	*R*	12 μm
Membrane	Thickness	*h*	~ 4 nm
Interior of vesicle	Conductivity	λ_in_	1.45 Sm^-1^
	Relative permittivity	ε_in_	70
Exterior of vesicle	Conductivity	λ_ex_	1.45 Sm^-1^
	Relative permittivity	ε_ex_	70
Membrane of vesicle	Conductivity	λ_m_	3.0×10^−7^ Sm^-1^
	Relative permittivity	ε_m_	4.5
	Resistivity	*ρ*_m_	3.3×10^6^ Ωm

The time dependent electric current module was used in the simulation where the parallel gold electrodes were selected. The numerical model demonstrated the importance of contact and angle between GUV and electrodes. For a certain value of electric potential with current density, the transmembrane voltage shows its threshold value for the rupture of targeted GUV.

### 2.4 Setup for applying the pulsed electric field on GUVs

**Fig 1:**
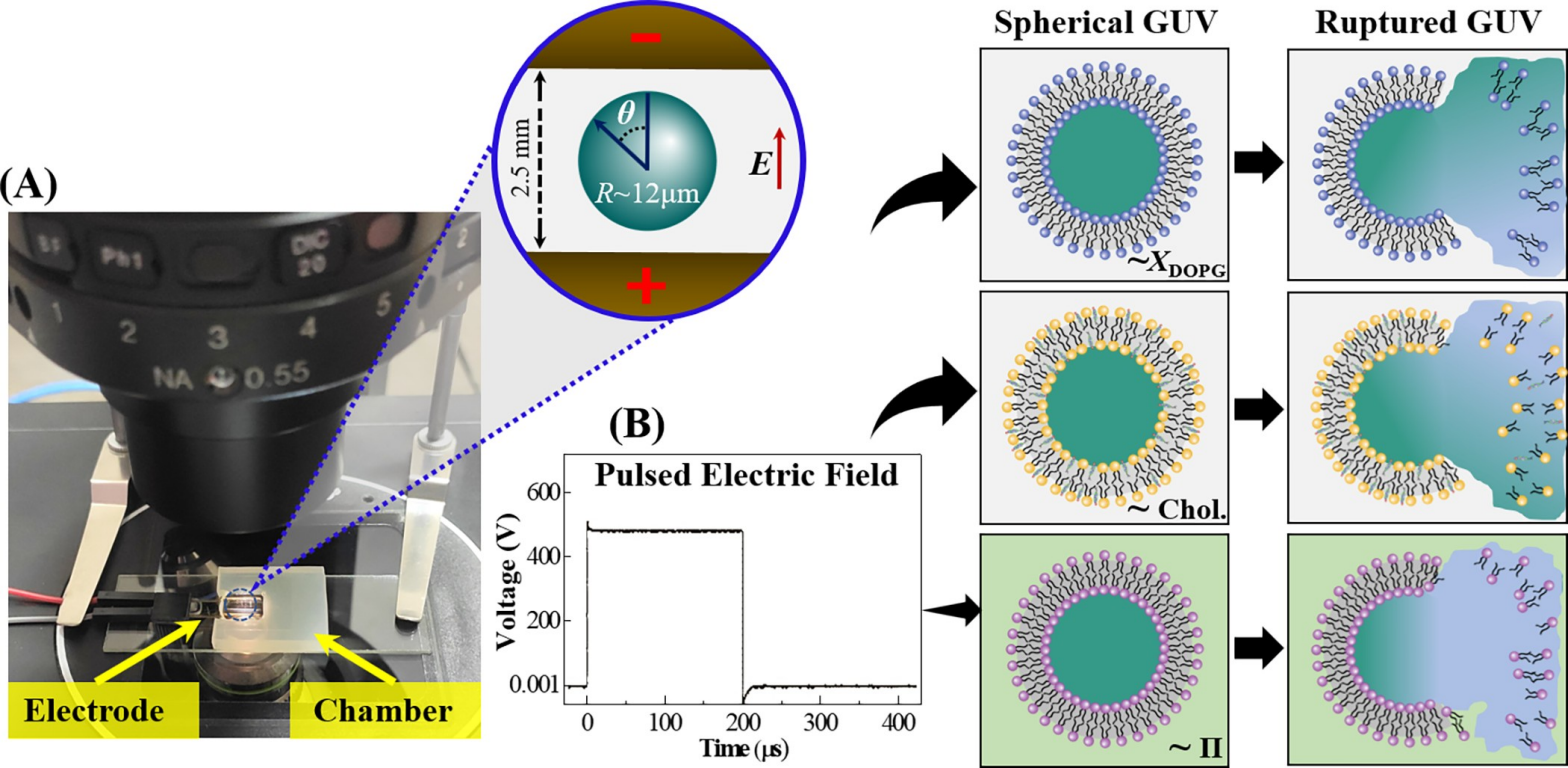
The electroporation processes. (A) Photograph of the electroporation setup with a scheme of a GUV between the electrodes (shown in the inset, not drawn to scale). (B) A pulsed electric field and the illustration of the electric field induced rupture of GUVs under DOPG lipid mole fraction (*X*_DOPG_), cholesterol (Chol) content and osmotic pressure (Π).

A MOSFET based IRE device was used for generating the pulsating DC (direct current) electric field with pulse width 200 μs and frequency 1.1 kHz. The detail circuit diagram was published in the earlier papers [22,63]. The photograph of IRE setup is shown in Fig 1(A). The IRE signal is applied to the GUV (GUV is kept in a U-shaped handmade microchamber) through gold coated electrodes of length 17.0 mm and width 2.54 mm (SH-17P-25.5, Hellotronics). The pulsed electric field, and the illustration of intact and ruptured vesicles under various conditions are shown in Fig 1(B).

### 2.5 Theoretical equations for applying pulsed electric field on GUVs

From the electrical point, transmembrane voltage (*V*_m_) is defined as the difference between the values of the homogeneous electric potential on both sides of the membrane. In the presence of pulsed electric field to a vesicle, when *V*_m_ exceeds ~ 1.0 V, the structural rearrangement of the lipid bilayer occurs, creating permanent aqueous pathways or pores in the membranes. The induced *V*m at each membrane point is defined as follows [64]:

$$V_{m}=f_{m}RE|\cos\theta |(1-e^{-t/\tau_{\mathrm{charg}}})$$ (2)

where, *R* is the radius of vesicle, *θ* is the angle between the electric field and surface potential and *τ*_charg_ is the membrane charging time constant. The expression for *f*_m_ is defined as follows [64]:

$$f_{m}=\frac{3\;\sigma_{\mathrm{ex}}[3\;h\;R^{2}\sigma_{\mathrm{in}}+(3\;h^{2}R-h^{3})(\sigma_{m}-\sigma_{\mathrm{in}})]}{2R^{2}(\sigma_{m}+2\sigma_{\mathrm{in}})(\sigma_{m}+\frac{1}{2}\sigma_{\mathrm{in}})-2{\left(R-h\right)}^{3}(\sigma_{\mathrm{ex}}-\sigma_{m})(\sigma_{\mathrm{in}}-\sigma_{m})}$$ (3)

where, the conductivity of membrane is *σ*_m_, internal medium is *σ*_in_, external environment is *σ*_ex_, and *h* is the thickness of membrane. Assuming *σ*_m_ = 0 (plasma membrane, *σ*_m_ = 3×10^−7^ Sm^-1^), then *f*_m_ = 1.5. Hence, Eq (3) is expressed as the first-order Schwan’s equation:

$$V_{m}=1.5RE|\cos\theta |(1-e^{-t/\tau_{\mathrm{charg}}})$$ (4)

where, *τ*_charg_ is the membrane charging-time, which is ~ 96.80 ns [22] for membrane capacitance, *C*_m_ ~ 1 μFcm^-1^ and GUV radius *R* = 10 μm. Therefore, Eq (4) can be written as follows:

$$V_{m}=1.5RE\cos\theta$$ (5)

The transmembrane voltage becomes maximum, when *θ* = 0. The rupture of GUVs occurs in that membrane site where transmembrane potential has a maximum value. The maximum value of *V*_m_ (= *V*_c_) is called the ‘critical transmembrane voltage for breakdown’ of vesicle. Then Eq (5) can be written as,

$$V_{m}=V_{c}=1.5RE$$ (6)

The value of *V*_c_ depends on the values of *R* and *E*, and in this experiment *V*_c_ ranges from 0.60 to 1.17 V.

### 2.6 Observation technique combined with high speed imaging

To observe the dynamics of vesicles under external electric field, the GUVs were visualized by an inverted phase contrast fluorescence microscope (Olympus IX-73, Japan) with a 20× objective. All experiments were performed at 25 ± 1°C. The images of GUVs were found from the recorded video using a charge-coupled device camera (Olympus DP22, Japan) with exposure time 111 ms. The frames per second (fps) of the camera was 25. A mercury lamp was used to acquire images of vesicles using the fast-digital camera. Phase contrast images were acquired using the cellSens Entry (Ver. 1.16) PC software (Olympus Corporation, Japan). The fluorescence intensity in the inside of GUVs was found from the active gray scale video using cellSens Dimension (Ver. 3.20) PC software (Olympus Corporation, Japan).

## 3 Results

### 3.1 Estimation of electric field for the rupture of GUVs using COMSOL simulation

**Fig 2:**
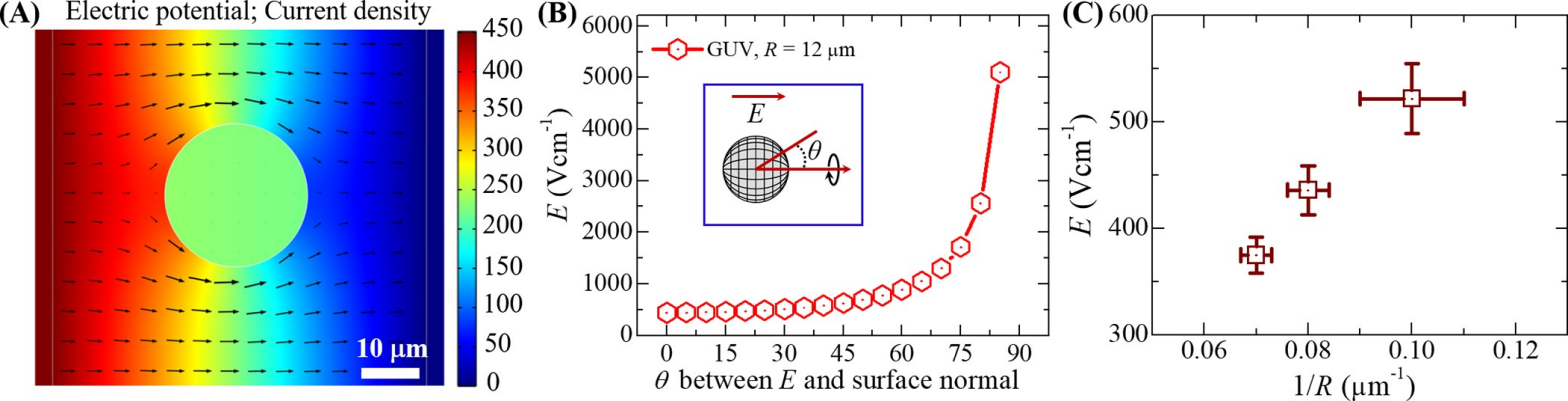
Electric field (*E*) strength for the permeabilization of spherical shaped GUV. (A) Distribution of current density with surface potential for lipid vesicle. The side color bar indicates the applied electric potential. (B) Electric field as a function of *θ* for *R* = 12 μm. (C) The 1/*R* dependent electric field for the rupture of GUVs.

Before going to quantify the electric field required for the rupture of vesicles, it was important to perform the simulation using the similar condition as used in the experimental study. It is mentioned earlier (see section 2.4) that the finite element-based software COMSOL Multiphysics is used to conduct the simulation. Fig 2(A) shows a simulation result in which the electric field was applied to a ‘single GUV’. In this case, 450 V potential was applied to the left sided gold coated electrode while the right sided electrode acted as ground. It is to be noted that for a certain value of electric potential with current density, the transmembrane voltage must be given at its threshold value (*V*_c_) for the rupture of GUVs. It was simulated the value of *V*_c_ for *R* = 12 μm GUV using Eq (6). The values of electric field with corresponding *θ* (*θ* is the angle between *E* and surface normal) is shown in Fig 2(B). It is very clear that the electric field required for the rupture of GUVs increases with the increase of *θ*. Moreover, all these values of *E* are responsible for rupturing of GUVs by creating pores in the membranes. If *θ* = 0, the rupture occurs at *E* ~ 450 Vcm^-1^, while higher values of *E* are required for the rupture of GUVs if *θ* changes from 0 to 90°. The value of electric field required for the rupture of GUVs is also dependent on the sizes of GUVs for a particular value of *θ*. As for example, *E* varied from 350 to 540 Vcm^-1^ for *R* = 10 to 15 μm at *θ* = 0. The 1/*R* dependent electric field required for vesicle rupture is shown in Fig 2(C) for the case of *θ* = 0. The value of *E* increases almost linearly with 1/*R*. This simulation work provided an important information on how much electric fields are needed for the rupture of GUVs in various experimental conditions.

### 3.2 Effect of pulsed electric field on the average time of rupture of GUVs

**Fig 3:**
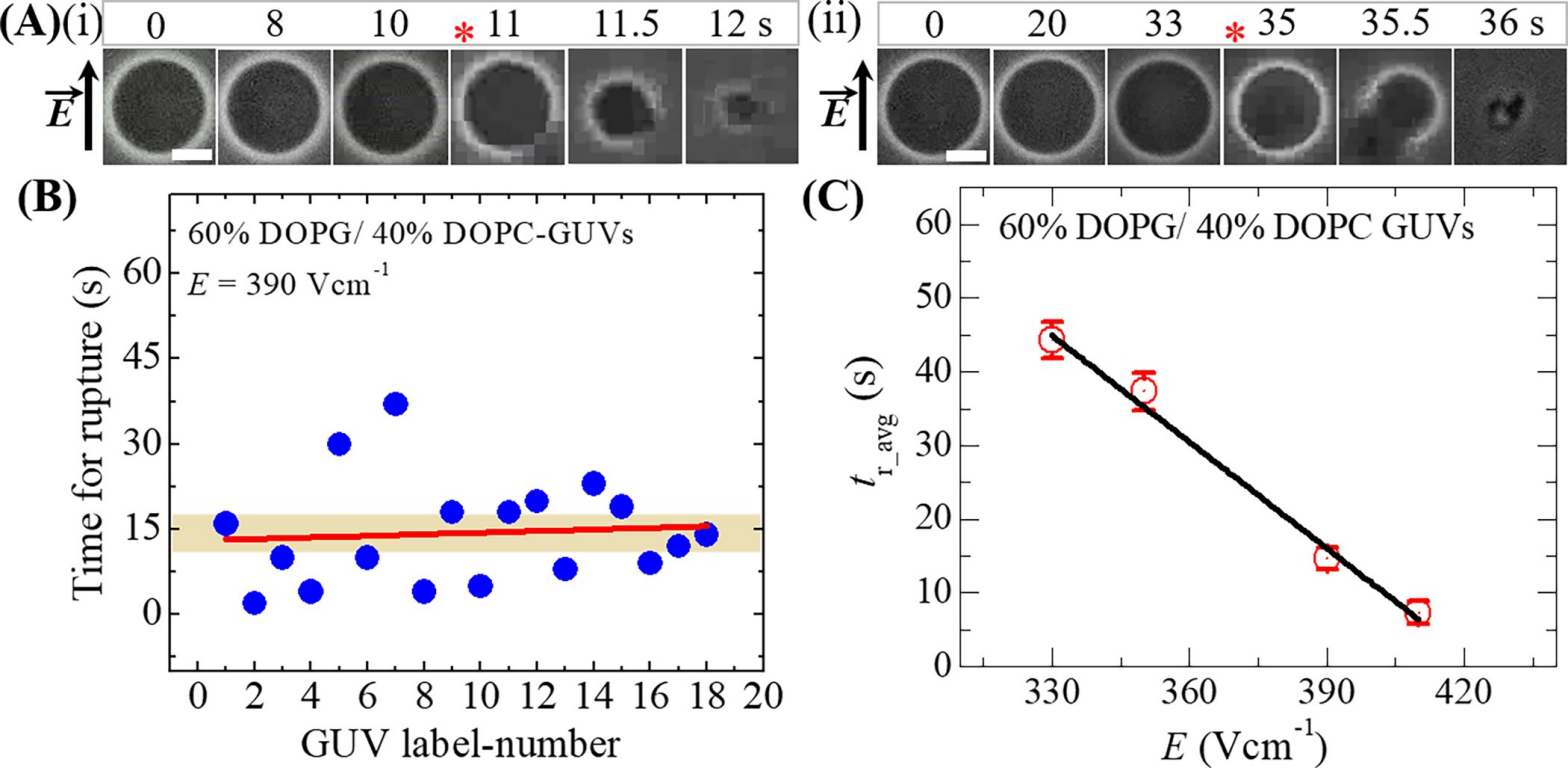
Stochastic rupture of several ‘single GUVs’ with phase contrast images and average rupture time after applying pulsed electric fields on the GUVs. (A) Phase contrast images of rupture of (i) first and (ii) second ‘single DOPG/DOPC (60/40)-GUV’ at *E* = 390 Vcm^-1^. The field direction is shown with an arrow in the left side. The numbers above in each image indicate the time in seconds after application of electric field. The time of rupture is indicated by the asterisk (*). The white bar corresponds to a length of 10 μm. (B) The time of stochastic rupture in several single DOPG/DOPC (60/40)-GUVs (number = 18) at *E* = 390 Vcm^-1^. (C) The *E* dependent average rupture time for DOPG/DOPC (60/40)-GUVs. The average values (*t*_r_avg_) and standard deviations are obtained using 3 independent experiments, each with 15˗24 GUVs for each *E*.

Here, the effect of pulsed electric field on a ‘single GUV’ has been investigated experimentally. In this case, an electric field, *E* = 390 Vcm^-1^ was applied on a ‘single DOPG/DOPC (60/40)-GUV’ (DOPG mole fraction *X*_DOPG_ = 0.60) for a maximum time 60 s. At time *t* = 0 s (i.e., before applying *E* due to electroporation signal), the GUV shows spherical structure in an inverted phase contrast image (Fig 3A(i)), and the structure remains unchanged until 10 s. The initiation of rupture starts at 11 s and subsequently the GUV is broken (Fig 3A(i)). Such rupture occurs due to the formation of pore in the membranes of vesicles as explained earlier [43,65]. The time of rupture is defined as the time when the vesicles start to rupture. We performed the similar experiment for 15−24 ‘single GUVs’. As for the presentation, we show only two ‘single’ GUVs’. Fig 3A(ii) represents the rupture of the 2^nd^ GUV, in which it occurs at 35 s. The rupture of several ‘single GUVs’ follows stochastic nature as shown in Fig 3(B), which means that the rupture of 18 GUVs occurs stochastically at different times in the presence of a fixed electric field (i.e., *E* = 390 Vcm^-1^). We calculated the average time (*t*_r_avg_) of the stochastic rupture by fitting the data. The solid red line (see Fig 3(B)) shows the linearly fitted curve from where *t*_r_avg_ = 14.4 ± 4.7 s is obtained. The similar experiment for rupture was done for *E* = 330, 350 and 410 Vcm^-1^ and calculated the *t*_r_avg_ in each condition. The value of *t*_r_avg_ decreases with the increase of *E* as shown in Fig 3(C). For *E* = 330 Vcm^-1^, *t*_r_avg_ = 44.33 ± 2.52 s and for *E* = 410 Vcm^-1^, *t*_r_avg_ = 7.33 ± 1.53 s.

The *E* dependent *t*_r_avg_ (s) data is fitted using a linear equation, and we obtain *t*_r_avg_ ~ 0 s when *E* ~ 420 Vcm^-1^. These investigations give us the information that how applied electric field influences the average time of the vesicle rupture. Interestingly, the values of external electric fields required for rupture of GUVs are very much consistent with the result as obtained in COMSOL simulation (see section 3.1).

### 3.3 Time of applied electric field dependent probability of rupture of GUVs

**Fig 4:**
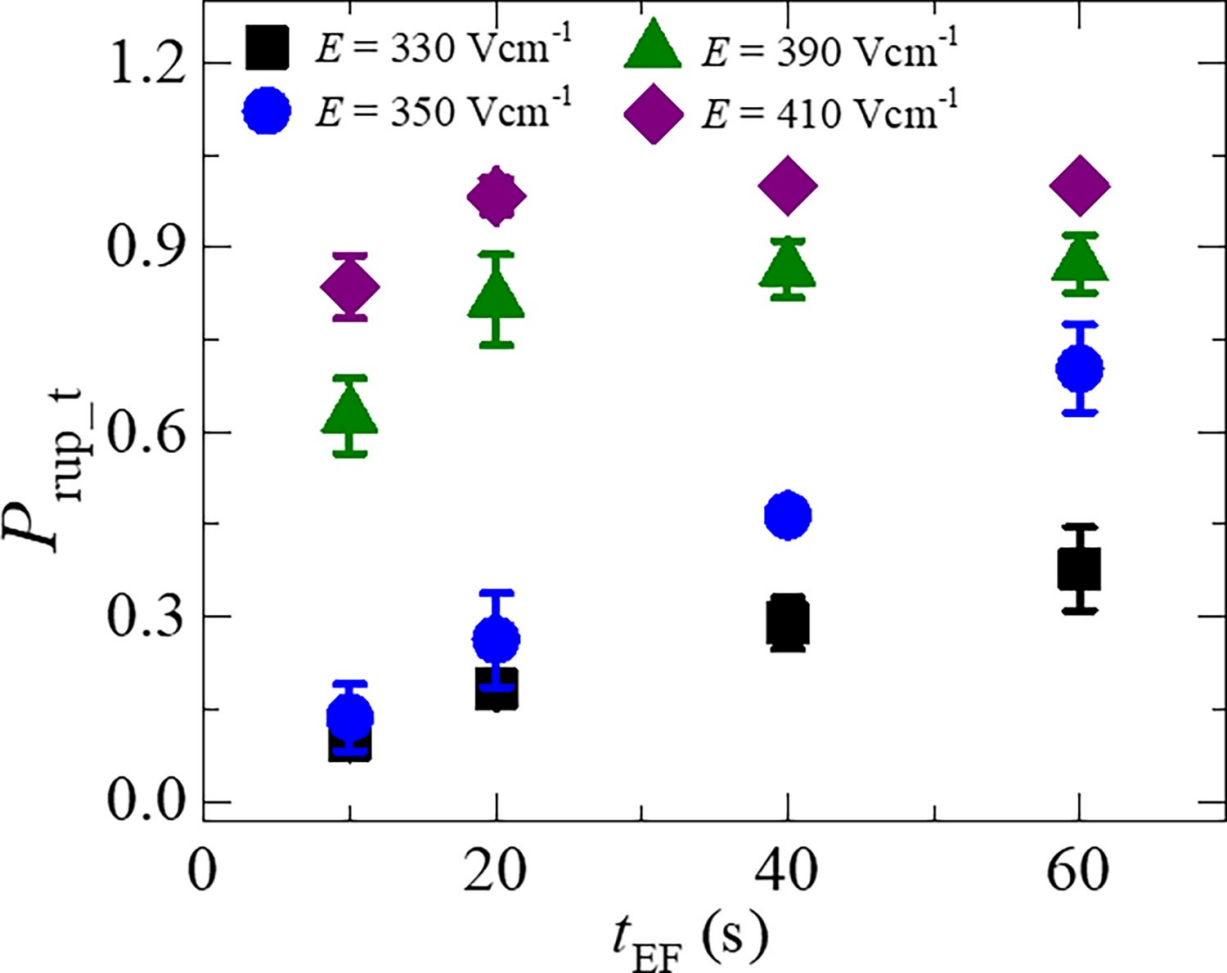
The time of applied electric field dependent probability of rupture of DOPG/DOPC (60/40)-GUVs until different time slots. Average and standard deviation are calculated from 3 independent experiments using 15˗24 GUVs for each case.

In this section, we experimentally investigated the probability of rupture (*P*_rup_t_) until different times (*t*_EF_) during the application of *E*. Fig 4 shows the dependence of *P*_rup_t_ of DOPG/DOPC (60/40)-GUVs for *E* = 330 Vcm^-1^ (◼) until the time, *t*_EF_ = 10, 20, 40 and 60 s. The probability of rupture is defined as the number of ruptured GUVs divided by the total number of observed GUVs. Suppose a number of 20 GUVs are investigated in 20 individual microchamber till a particular time after applying a specific electric field. If 10 GUVs are ruptured, *P*_rup_t_ = 10/20 = 0.5. At first, we observe the results of *E* = 330 Vcm^-1^ in which the value of *P*_rup_t_ increases with the increase of *t*_EF_. At 10 s, *P*_rup_t_ = 0.10 ± 0.01 and at 60 s, *P*_rup_t_ = 0.38 ± 0.07. Similar experiments were done for *E* = 350 Vcm^-1^ (●), *E* = 390 Vcm^-1^ (▲) and *E* = 410 Vcm^-1^ (◆) at the same time slot. In all conditions of electric field, the *P*_rup_t_ increases with *t*_EF_.

However, at a particular time slot, the value of *P*_rup_t_ is higher for higher electric field. As for example, at *t*_EF_ = 10 s, *P*_rup_t_ = 0.14 ± 0.05 for *E* = 350 Vcm^-1^ and 0.86 ± 0.05 for *E* = 410 Vcm^-1^. Moreover, at *E* = 410 Vcm^-1^, *P*_rup_t_ = 1.0 at 40 s and above. These investigations clearly show that the values of electric field required for rupture of GUVs are dependent on *t*_EF_.

### 3.4 Quantity of electric field for the rupture of GUVs containing various surface charges

**Fig 5:**
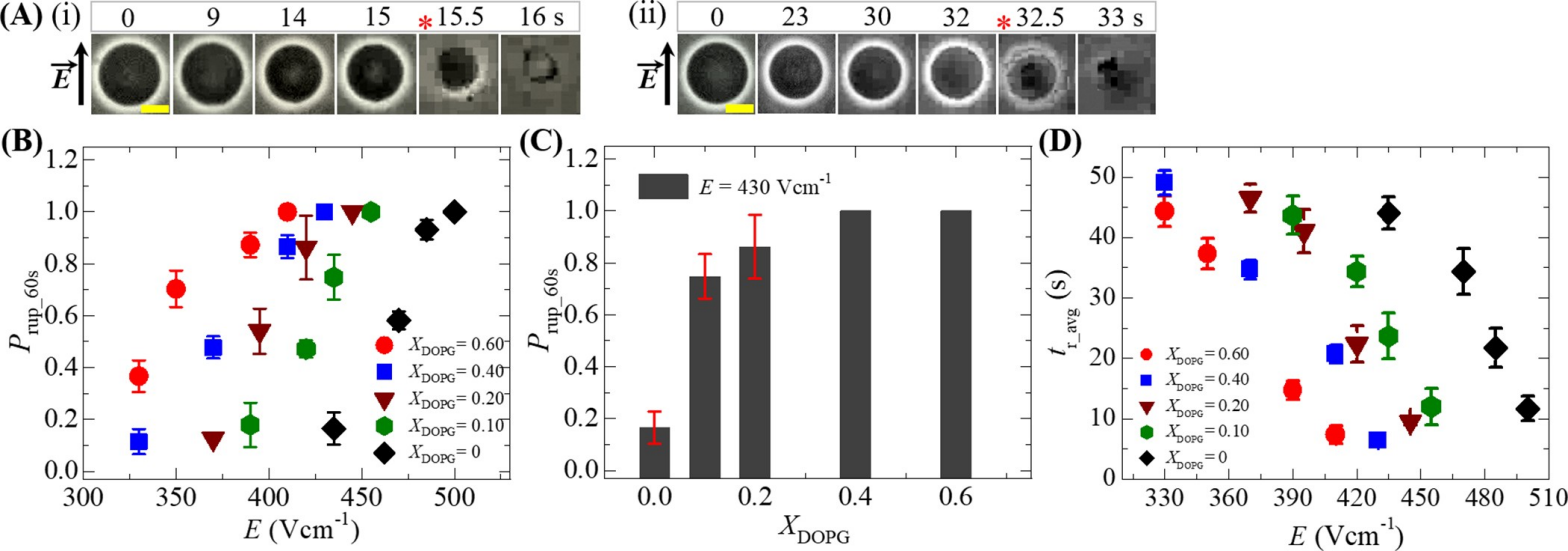
Electric field dependent probability of rupture and the average time of rupture of GUVs containing various surface charges in their membranes. (A) Phase contrast images of rupture of (i) a ‘single DOPG/DOPC (40/60)-GUV’ at *E* = 420 Vcm^-1^ and (ii) a ‘single DOPG/DOPC (10/90)-GUV’ at *E* = 420 Vcm^-1^. The field direction is shown with an arrow in the left side. The numbers above in each image indicate the time in seconds after application of electric field. The time of rupture is indicated by the asterisk (*). The yellow bar corresponds to a length of 10 μm. (B) The electric field dependent *P*_rup_60s_ for *X*_DOPG_ = 0.60 (●), 0.40 (◼), 0.20 (▼), 0.10 (⬢) and 0 (◆). (C) The *X*_DOPG_ dependent *P*_rup_60s_ at *E* = 430 Vcm^-1^. (D) The electric field dependent average rupture time for *X*_DOPG_ = 0.60 (●), 0.40 (◼), 0.20 (▼), 0.10 (⬢) and 0 (◆). Average and standard deviation are calculated from 3 independent experiments using 15˗24 GUVs for each case.

Here, we determine the electric field required for the rupture of GUVs prepared by various anionic charges in their membranes. The anionic charge was varied by changing the DOPG mole fraction (*X*_DOPG_) at 162 mM salt concentration in buffer. Electric field *E* = 420 Vcm^-1^ was applied on DOPG/DOPC (40/60)-GUV (here *X*_DOPG_ = 0.40) for a maximum time 60 s. The phase contrast image of a spherical shaped GUV is shown in Fig 5A(i), which was intact until *t* = 15 s and then ruptured at *t* = 15.5 s. The same electric field was also applied on DOPG/DOPC (10/90)-GUV (here *X*_DOPG_ = 0.10) and rupture occurred at *t* = 32.5 s (Fig 5A(ii)). Fig 5(B) shows the dependence of probability of rupture until 60 s, *P*_rup_60s_, for *X*_DOPG_ = 0.60 (●), 0.40 (◼), 0.20 (▼), 0.10 (⬢) and 0 (◆) for various *E*. In all cases of *X*_DOPG_, the value of *P*_rup_60s_ increases with *E*. However, as the value of *X*_DOPG_ decreases from 0.60 to 0, the electric field required for the similar value of *P*_rup_60s_ is larger. As an example, *P*_rup_60s_ = 0.87 ± 0.05 for *X*_DOPG_ = 0.60 at *E* = 390 Vcm^-1^ whereas *P*_rup_60s_ = 0.86 ± 0.12 for *X*_DOPG_ = 0.20 at *E* = 430 Vcm^-1^. It has also been investigated the *X*_DOPG_ dependence of *P*_rup_60s_ for a specific electric field. Fig 5(C) shows the *P*_rup_60s_ with *X*_DOPG_ for *E* = 430 Vcm^-1^ in which *P*_rup_60s_ = 0.16 ± 0.06 at *X*_DOPG_ = 0 is much lower than *P*_rup_60s_ = 0.86 ± 0.12 at *X*_DOPG_ = 0.20. The value of *P*_rup_60s_ = 1.0 at *X*_DOPG_ = 0.40 and 0.60 for the same electric field.

In addition, we measured the average time of rupture (*t*_r_avg_) for *X*_DOPG_ = 0.60 (●), 0.40 (◼), 0.20 (▼), 0.10 (⬢) and 0 (◆) for various *E* as shown in Fig 5(D). The values of *t*_r_avg_ decrease with the increases of *E* for all *X*_DOPG_. As an example, for *X*_DOPG_ = 0.20, *t*_r_avg_ = 49.00 ± 2.00 s at *E* = 330 Vcm^-1^_,_ *t*_r_avg_ = 34.67 ± 1.53 s at *E* = 370 Vcm^-1^, *t*_r_avg_ = 20.67 ± 1.53 s at *E* = 410 Vcm^-1^ and *t*_r_avg_ = 6.33 ± 0.58 s at *E* = 430 Vcm^-1^. Similar *t*_r_avg_ can be found by decreasing the electric fields for various *X*_DOPG._ For better understanding, the value of *t*_r_avg_ = 34.67 ± 1.53 s at *E* = 370 Vcm^-1^ for *X*_DOPG_ = 0.40 is almost similar to *t*_r_avg_ = 34.33 ± 2.52 s at *E* = 420 Vcm^-1^ for *X*_DOPG_ = 0.10 and also *t*_r_avg_ = 34.33 ± 3.79 s at *E* = 470 Vcm^-1^ for *X*_DOPG_ = 0. These investigations suggest that the mechanical stability of vesicles becomes weaker at higher surface charges in membranes. The electric field dependent *P*_rup_60s_, *V*_c_ and *t*_r_avg_ for various *X*_DOPG_ are presented in Table 2.

**Table 2:** The electric field dependent probability of rupture and average time for various surface charges, cholesterol contents and osmotic pressures (Both *X*_DOPG_ = 0.60 and Chol = 0 are same membrane. Again, both *X*_DOPG_ = 0.40 and *X*_DOPG_ = 0.40 with Δ*C*^0^ = 0 mOsmL^-1^ are same membrane).

Electric field *E* (Vcm^-1^)	Critical transmembrane voltage, *V*_c_ (V)	Membrane composition	Probability of rupture, *P*_rup_60s_	Average time of rupture, *t*_r_avg_ (s)
410	0.77	*X*_DOPG_ = 0.60	1.0	7.33 ± 1.53
430	0.83	*X*_DOPG_ = 0.40		6.33 ± 0.58
445	0.84	*X*_DOPG_ = 0.20		9.47 ± 0.55
455	0.86	*X*_DOPG_ = 0.10		12.00 ± 3.00
500	0.94	*X*_DOPG_ = 0		11.67 ± 2.08
410	0.77	Chol = 0	1.0	7.33 ± 1.53
500	0.94	Chol = 0.15		9.33 ± 1.53
575	1.09	Chol = 0.29		12.33 ± 2.08
630	1.17	Chol = 0.40		13.00 ± 1.00
430	0.83	*X*_DOPG_ = 0.40, Δ*C*^0^ = 0 mOsmL^-1^	1.0	6.33 ± 0.58
370	0.71	*X*_DOPG_ = 0.40, Δ*C*^0^ = 13 mOsmL^-1^		8.67 ± 2.02
300	0.60	*X*_DOPG_ = 0.40, Δ*C*^0^ = 17 mOsmL^-1^		10.00 ± 2.65

### 3.5 Quantity of electric field for the rupture of GUVs containing various concentrations of cholesterol in their membranes

**Fig 6:**
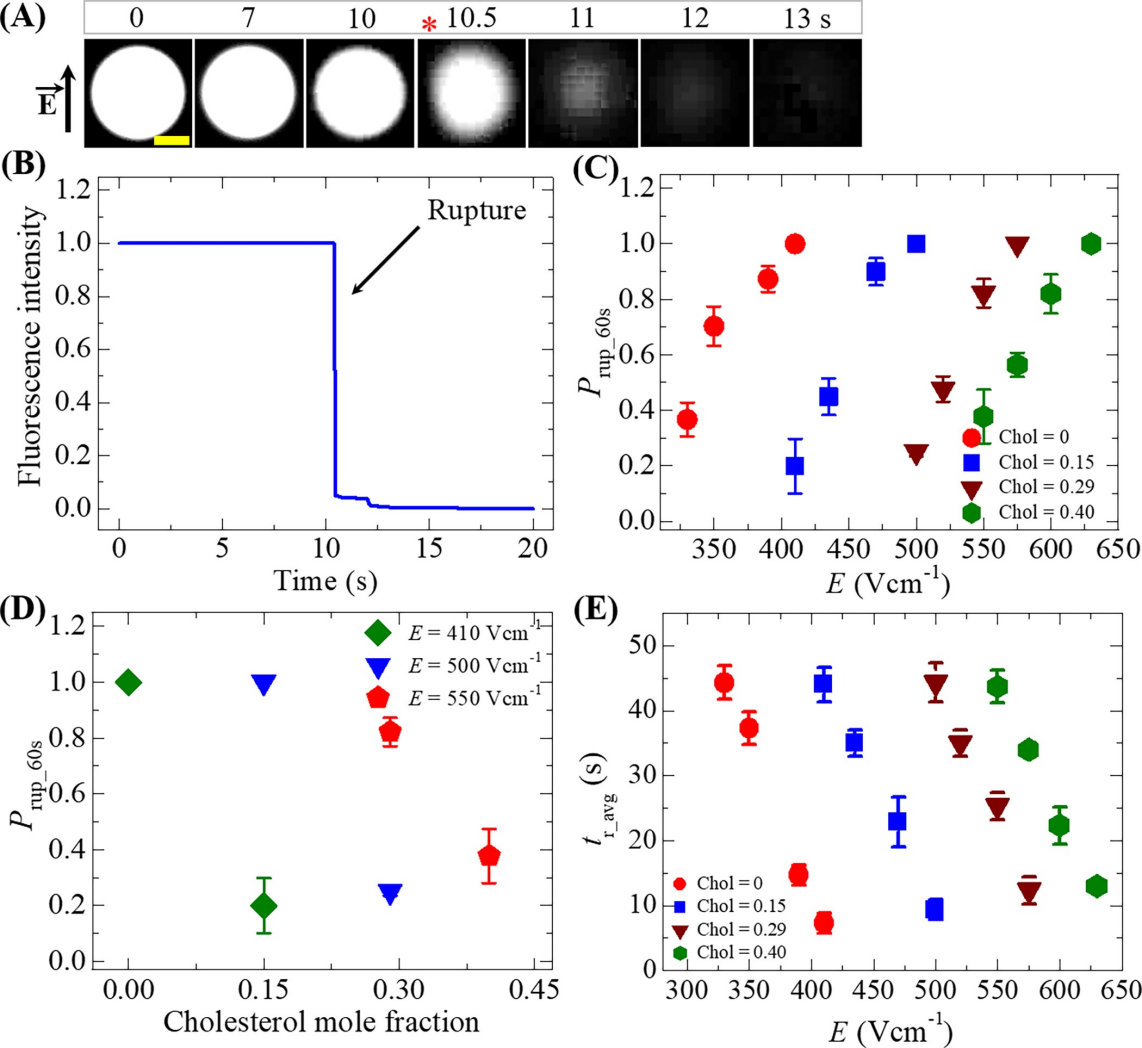
Electric field dependent probability of rupture and the average time of rupture of GUVs containing various cholesterol in their membranes. (A) Fluorescence images of rupture of a ‘single DOPG/DOPC/Chol (46/39/15)-GUV’ at *E* = 470 Vcm^-1^. The field direction is shown with an arrow in the left side of image. The numbers above in each image indicate the time in seconds after applying *E*. The yellow scale bar is 10 μm. The time of rupture is indicated by asterisk mark (*). (B) The time dependent normalized fluorescence intensity of GUV as shown in (a). (C) The electric field dependent *P*_rup_60s_ for Chol = 0 (●), 0.15 (◼), 0.29 (▼) and 0.40 (⬢). (D) The cholesterol dependent *P*_rup_60s_ at the values of *E* = 410 (◆), 500 (▼) and 550 Vcm^-1^ (⬟). (E) The electric field dependent average rupture time, *t*_r_avg_ for Chol = 0 (●), 0.15 (◼), 0.29 (▼) and 0.40 (⬢). Average and standard deviation are calculated from 3 independent experiments using 15˗24 GUVs for each case.

So far, we investigated how much electric field is required for the rupture of GUVs under various surface charges of membranes. Now the electric field is quantified for the rupture of vesicles containing various concentrations of cholesterol in their membranes. The value of *E* = 470 Vcm^-1^ was applied on a ‘single DOPG/DOPC/Chol (46/39/15)-GUV’ (here cholesterol mole fraction, Chol = 0.15) for a maximum time 60 s. In this case, the inside of vesicle was 1 mM calcein with 0.10 M sucrose. Prior to apply the pulsed electric field, the inside of GUV shows white color in a fluorescence microscopic image (Fig 6(A)) at 0 s due to this calcein solution. During the application of electric field, the spherical shaped GUV starts to rupture at 10.5 s and subsequently a complete rupture occurs at 13 s (Fig 6(A)). The time dependent fluorescence intensity of the same GUV is shown in Fig 6(B). This graph clearly indicates two-stage phenomena; one is intact state of GUV and another is rupture state. Moreover, it gives the exact time of the rupture of GUVs. The abruptly decreasing line of intensity denotes the rupture state. Fig 6(C) shows the dependence of *P*_rup_60s_ for Chol = 0 (●), 0.15 (◼), 0.29 (▼) and 0.40 (⬢) for various *E*. At a fixed cholesterol mole fraction, the value of *P*_rup_60s_ increases with the increase of *E*. However, as the cholesterol increases from 0 to 0.40, the electric field required for the similar *P*_rup_60s_ value is larger. As an example, *P*_rup_60s_ = 0.45 ± 0.07 for Chol = 0.15 at *E* = 435 Vcm^-1^ whereas *P*_rup_60s_ = 0.48 ± 0.05 for Chol = 0.29 at *E* = 520 Vcm^-1^.

Fig 6(D) shows the *P*_rup_60s_ with various cholesterols for *E* = 410, 500 and 550 Vcm^-1^. It is observed that at Chol = 0.15 the value of *P*_rup_60s_ = 0.20 ± 0.09 is much lower than *P*_rup_60s_ = 1.0. It means the *P*_rup_60s_ = 1.0 when *E* increases from 410 to 500 Vcm^-1^. Similar tendency is followed for other cholesterol concentrations. These investigations clearly show that as the cholesterol content increases in the membranes of vesicles, the mechanical stability become increases.

We also determine the average time of rupture (*t*_r_avg_) for Chol = 0 (●), 0.15 (◼), 0.29 (▼) and 0.40 (⬢) for various *E* (Fig 6(E)). The value of *t*_r_avg_ decreases with the increases of *E* for each Chol. As for example, *t*_r_avg_ = 44.00 ± 2.65 s at *E* = 410 Vcm^-1^_,_ *t*_r_avg_ = 35.00 ± 2.00 s at *E* = 435 Vcm^-1^, *t*_r_avg_ = 22.83 ± 3.82 s at *E* = 470 Vcm^-1^ and *t*_r_avg_ = 9.33 ± 1.53 s at *E* = 500 Vcm^-1^ for Chol = 0.15. The tendency of decreasing the value of *t*_r_avg_ with electric field for various cholesterol containing membranes is similar. The electric field dependent *P*_rup_60s_, *V*_c_ and *t*_r_avg_ for various cholesterol mole fraction are presented in Table 2.

### 3.6. Quantity of electric field for the rupture of GUVs under various osmotic pressures

**Fig 7:**
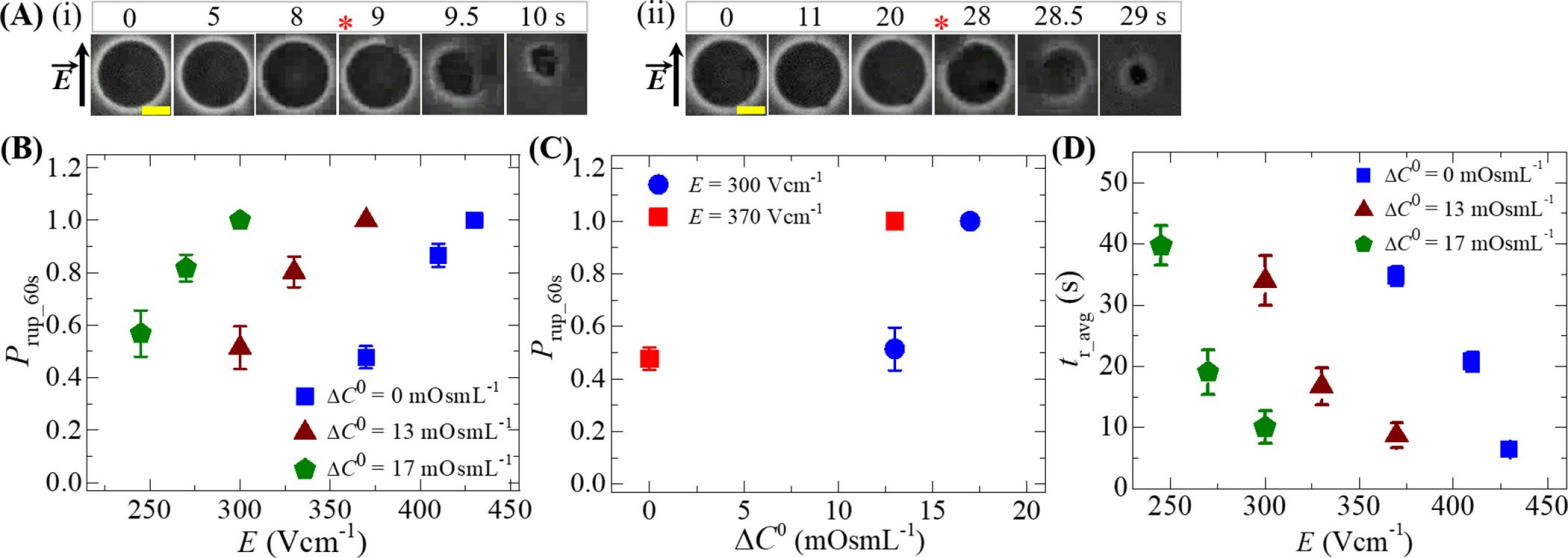
Electric field dependent probability of rupture and the average time of rupture of GUVs in the presence of various osmotic pressures. (A) Phase contrast images of rupture of (i) a ‘single DOPG/DOPC (40/60)-GUV’ at *E* = 300 Vcm^-1^ under 17 mOsmL^-1^ and (ii) a ‘single DOPG/DOPC (40/60)-GUV’ at *E* = 300 Vcm^-1^ under 13 mOsmL^-1^. The field direction is shown with an arrow in the left side. The numbers above in each image indicate the time in seconds after application of electric field. The time of rupture is indicated by the asterisk (•). The yellow bar corresponds to a length of 10 μm. (B) The electric field dependent *P*_rup_60s_ for Δ*C*^0^ = 0 (◼), 13 (▲) and 17 mOsmL^-1^ (⬟). (C) The Δ*C*^0^ dependent *P*_rup_60s_ at *E* = 370 (◼) and 300 Vcm^-1^ (●). (D) The electric field dependent *t*_r_avg_ for Δ*C*^0^ = 0 (◼), 13 (▲) and 17 mOsmL^-1^ (⬟). Average and standard deviation are calculated from 3 independent experiments using 15˗24 GUVs in each case.

The mechanical stability is greatly influenced by the surface charges and the cholesterol content in the membranes of vesicles as observed in sections 3.4 and 3.5. In this section, we quantify the electric field for the rupture of DOPG/DOPC (40/60)-GUVs under different osmotic pressures (Π). The osmolarity of the inside sucrose of GUVs,$C_{\mathrm{in}}^{0}$ was 388 mOsmL^-1^. When GUVs are transferred to a hypotonic solution of concentration *C*_out_ (mOsmL^-1^), osmotic pressure is induced in the GUV, which increases the radius of the membrane at swelling equilibrium. The osmolarity difference at an initial condition between the inside and the outside of GUV becomes $\Delta C^{0}=C_{\mathrm{in}}^{0}-C_{\mathrm{out}}$. We fixed the osmolarity of glucose solution *C*_out_ = 375 and 371 mOsmL^-1^, and hence the corresponding Δ*C*^0^ = 13 and 17 mOsmL^-1^. The detail description of maintaining the osmolarity difference is reported earlier [60]. The phase contrast images of GUV at *E* = 300 Vcm^-1^ under 17 and 13 mOsmL^-1^ are shown in Fig 7A(i) and 7A(ii), respectively. The corresponding GUVs became rupture at 9 and 28 s. Fig 7(B) shows the electric field dependent *P*_rup_60s_ for Δ*C*^0^ = 0 (◼), 13 (▲) and 17 mOsmL^-1^ (⬟). As the value of Δ*C*^0^ increases, the electric field required for the similar *P*_rup_60s_ is quite smaller. As an example, *P*_rup_60s_ = 0.87 ± 0.04 at Δ*C*^0^ = 0 for *E* = 410 Vcm^-1^ whereas *P*_rup_60s_ = 0.81 ± 0.06 at Δ*C*^0^ = 13 mOsmL^-1^ for *E* = 330 Vcm^-1^ and also *P*_rup_60s_ = 0.82 ± 0.05 at Δ*C*^0^ = 17 mOsmL^-1^ for *E* = 270 Vcm^-1^. In all conditions, the value of *P*_rup_60s_ increases with *E*. Fig 7(C) shows the *P*_rup_60s_ for *E* = 370 (◼) and 300 Vcm^-1^ (●) at different Δ*C*^0^. It is found that for *E* = 300 Vcm^-1^, *P*_rup_60s_ = 0.48 ± 0.04 at Δ*C*^0^ = 0 is much lower than *P*_rup_60s_ = 1.0 at Δ*C*^0^ = 13 mOsmL^-1^. Besides, *P*_rup_60s_ = 1.0 can be obtained by decreasing *E* from 370 to 300 Vcm^-1^ at Δ*C*^0^ = 13 mOsmL^-1^ (Fig 7(C)).

The value of *t*_r_avg_ for Δ*C*^0^ = 0 (◼), 13 (▲) and 17 mOsmL^-1^ (⬟) for various *E* are shown in Fig 7(D). The *t*_r_avg_ decreases with the increases of *E* for each Δ*C*^0^. As for example, *t*_r_avg_ = 34.00 ± 4.00 s at *E* = 300 Vcm^-1^_,_ *t*_r_avg_ = 16.67 ± 3.06 s at *E* = 330 Vcm^-1^ and *t*_r_avg_ = 8.67 ± 2.02 s at *E* = 370 Vcm^-1^ under Δ*C*^0^ = 13 mOsmL^-1^. However, the *t*_r_avg_ = 20.67 ± 1.53 s at *E* = 410 Vcm^-1^ for Δ*C*^0^ = 0 is similar to the value of *t*_r_avg_ = 19.00 ± 3.61 s at *E* = 270 Vcm^-1^ for Δ*C*^0^ = 17 mOsmL^-1^. These investigations suggest that a lower electric field is required for the rupture of GUVs for higher osmotic pressure. The electric field dependent *P*_rup_60s_, *V*_c_ and *t*_r_avg_ for various osmotic pressures are presented in Table 2.

## 4 Discussion

At first, we performed the COMSOL simulation for estimating the electric field required for the vesicle rupture. Then, we quantify the electric field for different conditions. The rupture of vesicles occurs when the transmembrane voltage is reached to a threshold value (*V*_c_), that is, when the externally applied electric field is above the electroporation threshold value. Electric field distribution, which is established in lipid membranes when electric current passes through the GUV, is difficult to predict, especially when the targeted vesicle has different conditions. For effective prediction of electric field strength and distribution, we performed the simulation study using the similar experimental condition (Fig 2). The electroporation depends on the effective electric field, which is directly related to the angle between the field and the membrane surface normal. The simulation results also show that the critical electric field strength for vesicle rupture depends linearly on reciprocal of the radius of GUVs, which agrees with Eq (5).

In the experimental study, for getting *P*_rup_60s_ = 1.0, the value of *E* changes from 500 to 410 Vcm^-1^ by changing the *X*_DOPG_ from 0 to 0.60. On the other hand, for getting the same *P*_rup_60s_, the value of *E* changes from 410 to 630 Vcm^-1^ by changing the cholesterol mole fraction from 0 to 0.40. In addition, the value of *E* changes from 430 to 300 Vcm^-1^ by changing the Δ*C*^0^ from 0 to 17 mOsmL^-1^. A numerical calculation regardless of the vesicle geometry was investigated by applying the electric field 600 Vcm^-1^ in which *V*_c_ was 0.80 V [66]. In our simulation, the electric field for rupture of GUV is obtained 350 to 540 Vcm^-1^ at *θ* = 0, which support that numerical calculation.

The coarse-grained MARTINI force field simulations indicated the instability of lipid membranes at higher electric fields [67]. With the increase of electric field, the undulation amplitude increases and consequently decreases the membrane density, which leads to the formation of pores in the membranes of GUVs. Such pores appear at the highly curved regions of the membranes. The formation of pore reorients the water-bilayer interface. The driving mechanism for the instability of membranes is related to the well-known fact that, when an electric field is applied, the low-energy configuration corresponds to the one where the interface aligns parallel to the applied field [68]. As the higher pulsed electric field creates instability of membranes, the electric field dependent average time for rupture (rupture occurs when the radius of pores increases to infinite within a very short time) of GUVs is lower, that supported our investigations (Fig 3(C)).

The intramembrane electrostatic effect due to the anionic lipids in the membranes destabilize the vesicles [69]. The electrostatic effect became prominent as the anionic charged lipids were added to the membranes [70–72]. Again, as the anionic lipid mole fraction increases in the membranes, the repulsive force between the lipid molecules also increases and hence increases the electrostatic effect [73]. With the increase of electrostatic interaction, the probability of rupture increases and consequently the average time of rupture decreases, supporting our investigation (Fig 5). Hence, membrane electrostatics play vital role for the processes of rupture of lipid vesicles.

Recently, it has been reported that as the cholesterol content increased in the membranes of DOPC vesicles, the bending rigidity increased several folds [59]. The results also indicated the local stiffening in DOPC membranes due to the addition of cholesterol. Buckling simulations on DOPC membranes also indicated the increase of bending rigidity [74]. Therefore, it can be reasonably considered that the membrane instability in cholesterol containing membranes due to electric field became lower compared to the membranes without cholesterol. Therefore, higher electric field is required for the rupture of GUVs as the cholesterol increases in the membranes that follows our investigations (Fig 6).

The electroporation in GUVs under various osmotic pressures was reported earlier [60], where we calculated the membrane tension generated by the osmotic pressures. In present report, we have aimed to quantify the pulsed electric field for the rupture of GUVs with various surface charges, cholesterols and osmotic pressures. It is well reported that the membrane tension due to electric field (*σ*_e_) is connected with transmembrane voltage (*V*_m_) [75,76] by $\sigma_{e}\infty V_{m}^{2}$. Since, *V*_m_∞*E*, hence *σ*_e_∞*E*^2^. Osmotic swelling creates lateral membrane tension (*σ*_os_) in the GUVs [77,78]. Therefore, the total tension in the membrane of GUVs is *σ*_t_ = *σ*_os_ + *σ*_e_ (if there is no osmotic effect, *σ*_t_ = *σ*_e_). With the increase of osmotic pressure, the value of *σ*_t_ increases, and therefore, the stability of membranes decreases. Again, the probability of rupture is defined as follows [79]:

$$P_{\mathrm{rup}\_t}=1-\exp(-k_{p}t_{\mathrm{EF}})$$ (7)

where, *k*_p_ is the rate constant of vesicle rupture. Generally, the rate constant for any reaction can be expressed by the following well-known Arrhenius equation [80]:

$$k_{p}=A\exp(-U_{b}/k_{B}T)$$ (8)

where, *A* is a constant whose unit is s^-1^, *k*_B_ is the Boltzmann constant and *T* is the absolute temperature. According to the classical theory of pore formation in lipid bilayers, the energy barrier of a prepore at a critical radius is defined as follows [79,81]:

$$U_{b}(r,\sigma_{t})=\frac{\pi \Gamma^{2}}{\sigma_{t}+B}$$ (9)

where, *B* is the electrostatic term for charged lipids in membranes [72], Γ is the line tension of a prepore and *r* is the prepore radius.

As the anionic lipid in membranes increases, the repulsive force between the anionic lipids becomes stronger. Such stronger effect increases the value of *B* and decreases the value of *U*_b_ (see Eq 9), which ultimately increases the probability of rupture (using Eqs 7 and 8) as obtained in Fig 5. Again, several reports indicated that the increase of cholesterol increase the value of Γ [82–85]. The higher is the value of Γ, the lower the value of *U*_b_, which eventually decreases the probability of rupture as found in Fig 6. Lastly, with the increase of osmotic gradient, the value of *σ*_os_ in *σ*_t_ increases. With the increased *σ*_os_, the value of *U*_b_ decreases, and consequently increases the rupture probability. This explanation is consistent with the result mentioned in Fig 7. Therefore, the above discussion supports our investigations.

## 5 Conclusions

We quantify the pulsed electric field required for the rupture of GUVs along with the behavior of such vesicles exposed to electric field. The optically detectable rupture is identified in the phase contrast and fluorescence images due to the disruption in membrane integrity. The amount of electric field required for vesicle rupture depends on the surface charges, cholesterol contents and the osmotic pressures. Addition of anionic lipid in membranes requires relatively lower electric field for the rupture of GUVs. In contrast, higher cholesterol content requires relatively higher electric field for vesicle rupture. Again, the value of applied electric field for vesicle rupture is greatly influenced by the osmotic gradient, indicating that higher gradient required lower electric field. Such differences are well explained by the mechanical stability of membranes of vesicles. The pore formation in the membranes of vesicles and consequently the rupture of vesicles are explained by the well accepted classical theory of pore formation in lipid bilayers. These investigations provide quantitative and valuable information on the electric field dependent rupture of GUVs under various conditions. This study serves as a guideline for further experiments in this area and offers an entrancing biophysical description of the phenomenon of electroporation in vesicles together with the insight of biological consequences.
